# Computerized tomography findings in calcified signet-ring gastric cancer receiving chemotherapy: a case report

**DOI:** 10.1186/s12885-018-4415-5

**Published:** 2018-04-27

**Authors:** Ting Wang, Zhi-Yong Lu, Xin-Fei Tu, Shui-Hong Zhang, Fang Huang, Long Huang

**Affiliations:** 1 Department of Oncology, The Second Afiliated Hospital of Nanchang University, 1 Minde Road, Nanchang, China; 2 JiangXi Key Laboratory of Clinical and Translational Cancer Research, Nanchang, Jiangxi China; 3 Department of Oncology, Nanchang county people’s hospital, Nanchang, China

**Keywords:** Gastric cancer, Calcification, Computerized tomography, Chemotherapy, Prognosis

## Abstract

**Background:**

Calcification in primary gastric cancer is very rare. In this report, we describe the computerized tomography (CT) changes in calcification in a patient with locally advanced signet-ring gastric cancer treated with chemotherapy.

**Case presentation:**

A 49-year-old man presented with 5 months’ history of abdominal pain, anorexia, and rapid weight loss. He had undergone Billroth-II subtotal gastrectomy for a bleeding gastric ulcer 30 years ago. Abdominal CT showed irregular thickening of the gastric wall and miliary calcifications. Histologic examination of specimen obtained by endoscopic biopsy showed poorly differentiated calcified signet-ring gastric cancer. The patient was clinically staged T4N2M0 and treated with docetaxel, cisplatin, and fluorouracil (DCF)/oxaliplatin and S-1 (XLOX)/S-1. After five cycles of chemotherapy, the general condition of the patient improved and tumor markers (CEA, CA125, CA199) decreased. However, follow-up CT scans showed continuing increase in the calcification.

**Conclusions:**

To conclude, in this case report we have described the dynamic changes in calcification in a gastric cancer patient receiving chemotherapy. One explanation for the observed increase in calcifications could be that the ischemic necrosis resulting from chemotherapy creates an alkaline environment, which promotes deposition of calcium salts. Our theory needs to be confirmed with histological evidence from a large series of patients. Nevertheless, we hope that these findings will improve understanding of the mechanism of calcification in gastric cancer.

## Background

Gastric cancer is one of the commonest malignancies [1]. Calcification in primary gastric cancer is uncommon and is seen in less than 3% of cases [2]; most of these tumors are adenocarcinomas containing pools of mucin.

In general, calcifications within malignancies are circumscribed and round in shape, with diameter ranging from 5 to 100 μm [3]. Calcifications may be seen in tumors of the thyroid, thymus, and pancreas, or in meningiomas, and are especially common in tumors of the female reproductive system (ovary, endometrium) and breast [3–8].

Gastric carcinoma with calcification characteristically occurs in relatively young patients [9, 10]. In most previous reports of calcified gastric cancer, the focus has been on imaging findings and pathological features; the patients generally received surgical resection and so the authors could not observe the progression of the calcification.

In this report, we present the serial computerized tomography (CT) features of a locally advanced calcified signet-ring cell gastric cancer that showed partial response to chemotherapy.

## Case presentation

**Fig. 1:**
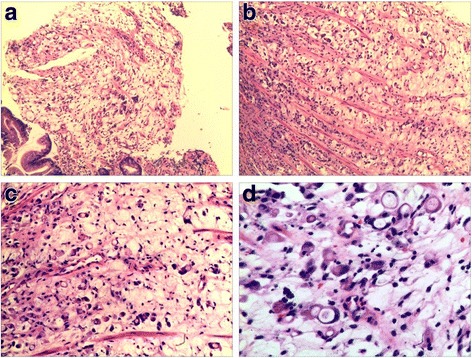
Histopathological examination shows signet-ring cells and psammoma bodies. A, B (× 100); C (× 200); D (× 400)

**Fig. 2:**
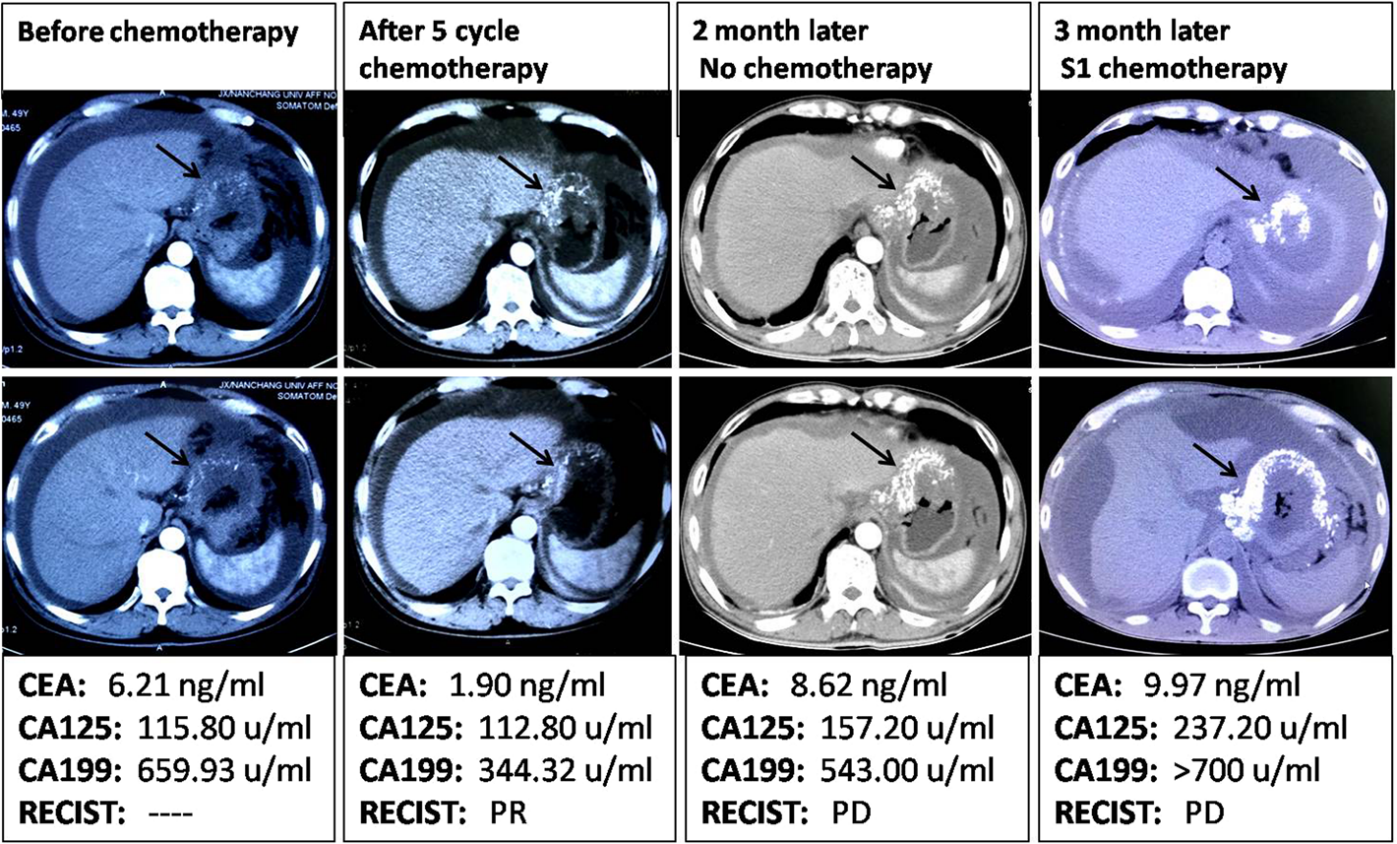
Serial abdominal CT scans show continuous increase in the calcifications (arrow) since starting chemotherapy

A 49-year-old man visited to our hospital in December 2016 with complaints of abdominal pain, anorexia, and rapid weight loss for 5 months. He had undergone Billroth-II subtotal gastrectomy 30 years earlier for treatment of a bleeding gastric ulcer. Abdominal CT scan revealed irregular thickening of the gastric walls and miliary calcifications. Histopathological examination of specimen obtained by endoscopic biopsy showed poorly differentiated signet-ring cell cancer with calcifications (Fig. 1). Tumor markers were elevated (CEA 6.21 ng/mL, CA125 115.80 u/mL, and CA199 659.93 u/mL). Serum calcium and phosphorus levels were within the normal range. The clinical stage was T4N2M0. A tumor at this stage is inoperable, and so the patient was started on chemotherapy with docetaxel, cisplatin, and fluorouracil (DCF). He received three cycles of DCF, but in March 2017 the chemotherapy protocol was changed to XLOX (oxaliplatin and S-1) because of toxic response. After five cycles of chemotherapy (3 cycles of DCF + 2 cycles of XLOX) the symptoms of abdominal pain and anorexia were relieved, and CEA, CA125, and CA199 decreased to 1.90 ng/mL, 112.80 u/mL, and 344.32 u/mL, respectively. Abdominal CT showed reduction in gastric wall thickening. However, the calcifications had increased **(**Fig. 2). The patient refused further chemotherapy and was lost to follow-up for 2 months.

In July 2017, the patient returned to the hospital with complaints of epigastric pain and abdominal distention. Abdominal CT showed progressive disease according to Response Evaluation Criteria in Solid Tumors (RECIST) criteria and increased calcifications (Fig. 2). The patient was started on palliative oral chemotherapy with S-1.

## Discussion and conclusions

Calcification in stomach cancer is rare, and there have been few reports since the initial description by Fukuda in 1922 [11]. Based on the imaging and pathologic characteristics, calcification can be of three types: mucous calcification, psammomatous calcification, or heterotropic ossification. Mucous calcification is seen within the mucin pools that are characteristic of advanced diffuse mucinous adenocarcinoma, whereas psammomatous calcification is seen within glandular lumina and stroma of non-mucin-producing intestinal carcinomas [12]. Heterotropic ossification refers to the calcification seen in primary and metastatic well-differentiated adenocarcinoma [10]. Of these, mucous calcification is relatively common. Almost all cases of mucous calcification are seen in adenocarcinomas or signet-ring cell carcinomas with large amounts of mucus secretion. The patient reported in this study also had signet-ring cell gastric cancer. The reason for the calcification in stomach cancer is still unclear. Psammomatous and non-psammomatous calcification may be induced by osteopontin protein produced by macrophages [13]. Murayama et al. have reported a case of gastric cancer that was found to produce a parathyroid hormone (PTH)-like substance; the authors suggested that this may have a role in the pathogenesis of psammomatous calcification [14]. The alkaline environment in the mucin pools may also promote calcification. Mucin is a similar cartilage calcification tendency of glycoprotein. Alkaline environment has accelerated the calcium salt deposits.

Whether the degree of calcification is related to the response to chemotherapy and prognosis is still unclear. Patients with mucinous gastric cancer and diffuse calcifications tend to be relatively young, and therefore survival may be better in them than in those having gastric cancer without calcification [10]. Among patients with serous adenocarcinoma of the ovary, those with calcification have significantly better survival than those without calcification. However, in papillary carcinoma of the thyroid the presence of calcification and the number of calcifications were not found to have any prognostic significance [15]. The significance of calcification in gastric adenocarcinoma is not known.

The general condition of our patient improved after five cycles of chemotherapy: symptoms subsided and abdominal CT and serum tumor markers showed improvement; however, the amount of calcification increased. Luca et al. reported a patient with calcified signet-ring cell gastric cancer in whom the calcification decreased gradually following chemotherapy. However, in our patient, serial abdominal CT scans showed continuous increase in the calcifications since starting chemotherapy. This may have been because of dystrophic calcification in chemotherapy-induced areas of ischemic necrosis. Mainly due to protein denaturation after more combined with phosphate, and the calcium ions internal flow after cellular damage form calcium phosphate [16]. Another possibility is that chemotherapy created an alkaline environment in the tumor [17]. Chemotherapy results in areas of ischemia and necrosis. Poor blood supply leads to decrease in cellular respiration and carbon dioxide production, which results in a relative alkalinity. Calcium salts, being poorly soluble in alkaline solutions, get deposited in the tissues [17, 18].

To conclude, in this case report we have described the dynamic changes in calcification in a gastric cancer patient receiving chemotherapy. One explanation for the observed increase in calcifications could be that the ischemic necrosis resulting from chemotherapy creates an alkaline environment, which promotes deposition of calcium salts. Our theory needs to be confirmed with histological evidence from a large series of patients. Nevertheless, we hope that these findings will improve understanding of the mechanism of calcification in gastric cancer.

## Abbreviations

CA125: Carbohydrate antigen 125
CA199: Carbohydrate antigen 199
CEA: Carcinoembryonic antigen
CT: Computerized tomography
DCF: Docetaxel + cisplatin + fluorouracil
RECIST: Response evaluation criteria in solid tumors
S-1: Tegafur-gimeracil-oteracil potassium capsule
XLOX: Oxaliplatin + S-1

## References

[1] Siegel RL, Miller KD, Jemal A (2017). Cancer statistics, 2017. CA Cancer J Clin.

[2] Park MS, Yu JS, Kim MJ, Yoon SY, Kim SH, Noh TW (2002). Mucinous versus nonmucinous gastric carcinoma: differentiation with helical CT. Radiology.

[3] Olsen JL, Penney DP, Averill KA (1977). Fine structural studies of a human thyroid adenoma, with special reference to psammoma bodies. Hum Pathol.

[4] Johannessen JV, Sobrinho-Simoes M (1980). The origin and significance of thyroid psammoma bodies. Lab Investig.

[5] Kepes J (1961). Electron microscopic studies of meningiomas. Am J Pathol.

[6] Budka H (1982). Hyaline inclusions (pseudopsammoma bodies) in meningiomas: immunocytochemical demonstration of epithel-like secretion of secretory component and immunoglobulins a and M. Acta Neuropathol.

[7] Cameron RI, McCluggage WG (2004). Extensive psammomatous calcification of the uterus and cervix associated with a uterine serous carcinoma. J Clin Pathol.

[8] Frenczy A, Talens M, Zoghby M, Hussain SS (1977). Ultrastructural studies on morphogenesis of psammoma bodies in ovarian serous neoplasia. Cancer.

[9] Niwa Y, Goto H, Hayakawa T (1998). Early gastric cancer with psammomatous calcification. Hepato-Gastroenterology.

[10] Balestreri L, Canzonieri V, Morassut S (1997). Calcified gastric cancer-CT findings before and after chemotherapy. Case report and discussion of the pathogenesis of this type of calcification. Clin Imaging.

[11] Fukuda T (1922). A case of adenocarcinoma of the stomach with deposit of calcified granules. Iji shinbun (Med News).

[12] Imai T, Murayama H, Arima S (1979). Heterotopic ossification and psammomatous calcification in gastric carcinoma: case report and review of literature. Acta Pathol Jap.

[13] Kawahara K, Niguma T, Yoshino T (2001). Gastric carcinoma with psammomatous calcification after Billroth II reconstruction: case report and literature review. Pathol Int.

[14] Murayama H, Kamio A, Imai T (1982). Gastric carcinoma with psammomatous calcification: report of case with reference to calculogenesis. Cancer.

[15] Rosai J, Carcangiu ML, DeLellis RA (1992). Papillary carcinoma. In: atlas of tumor pathology, 3rd series, fascicle 5, “tumor of the thyroid”.

[16] Gutiérrezd OA, Asteinza M, Loscos JM (2001). Endoscopic ultrasonography features of calcified gastric cancer. Hepato-gastroenterol.

[17] Rotondo A, Grassi R, Smaltino F (1986). Calcified gastric cancer: report of a case and review of literature. Brit J Radiol.

[18] D'Altorio RA (1973). Calcification in a gastric mucinous adenocarcinoma. Am J Dig Dis.

